# Ischemic Tolerance Induced by Glial Cells

**DOI:** 10.1007/s11064-022-03704-y

**Published:** 2022-08-03

**Authors:** Schuichi Koizumi, Yuri Hirayama

**Affiliations:** 1 Department of Neuropharmacology, Yamanashi, Japan; 2 Yamanashi GLIA Center, Interdisciplinary Graduate School of Medicine, University of Yamanashi, 409-3898 Yamanashi, Japan; 3 Department of Pharmacology, Graduate School of Medicine, Chiba University, 260-8670 Chiba, Japan

**Keywords:** Ischemic tolerance, Microglia, Astrocytes, Purinergic signals, P2X 7 receptor.

## Abstract

Ischemic tolerance is a phenomenon in which resistance to subsequent invasive ischemia is acquired by a preceding noninvasive ischemic application, and is observed in many organs, including the brain, the organ most vulnerable to ischemic insult. To date, much research has been conducted on cerebral ischemic tolerance as a cell-autonomous action of neurons. In this article, we review the essential roles of microglia and astrocytes in the acquisition of ischemic tolerance through neuron-non-autonomous mechanisms, where the two types of glial cells function in a concerted manner to induce ischemic tolerance.

## Background of Stroke Therapy

The brain is the organ most vulnerable to ischemic insult. There have been more than 1,000 clinical trials of brain-protective drugs that target neurons to treat stroke and improve prognosis, but few have had sufficient therapeutic efficacy [1]. In this context, the late Dr. Barres, one of the leaders in brain science research stated “Glial cells know how to save the brain, but brain scientists don’t know it yet,” an ironic reference to the state of current brain science, which is biased toward neuronal research [2].

Stroke, the second most serious cause of death worldwide, is a medically and socially important disease because it is often associated with significant sequelae even after a patient survives. Indeed, stroke is the number one causative disease that requires extensive care. Stroke can be broadly classified as cerebral infarction, in which a blood vessel becomes clogged, and cerebral hemorrhage or subarachnoid hemorrhage, in which a blood vessel ruptures, with cerebral infarction accounting for the highest percentage of cases. Securing blood flow is the most important treatment strategy, and the development and improvement of thrombolytic therapy using tissue plasminogen activator (tPA) have greatly advanced treatment [3]. However, there are still many unresolved issues related to its therapeutic use, such as the limited time window for the indication of tPA therapy and the inability to address so-called delayed neuronal cell death, in which neuronal damage and death continue to progress even after the blood flow is restored.

**Fig. 1:**
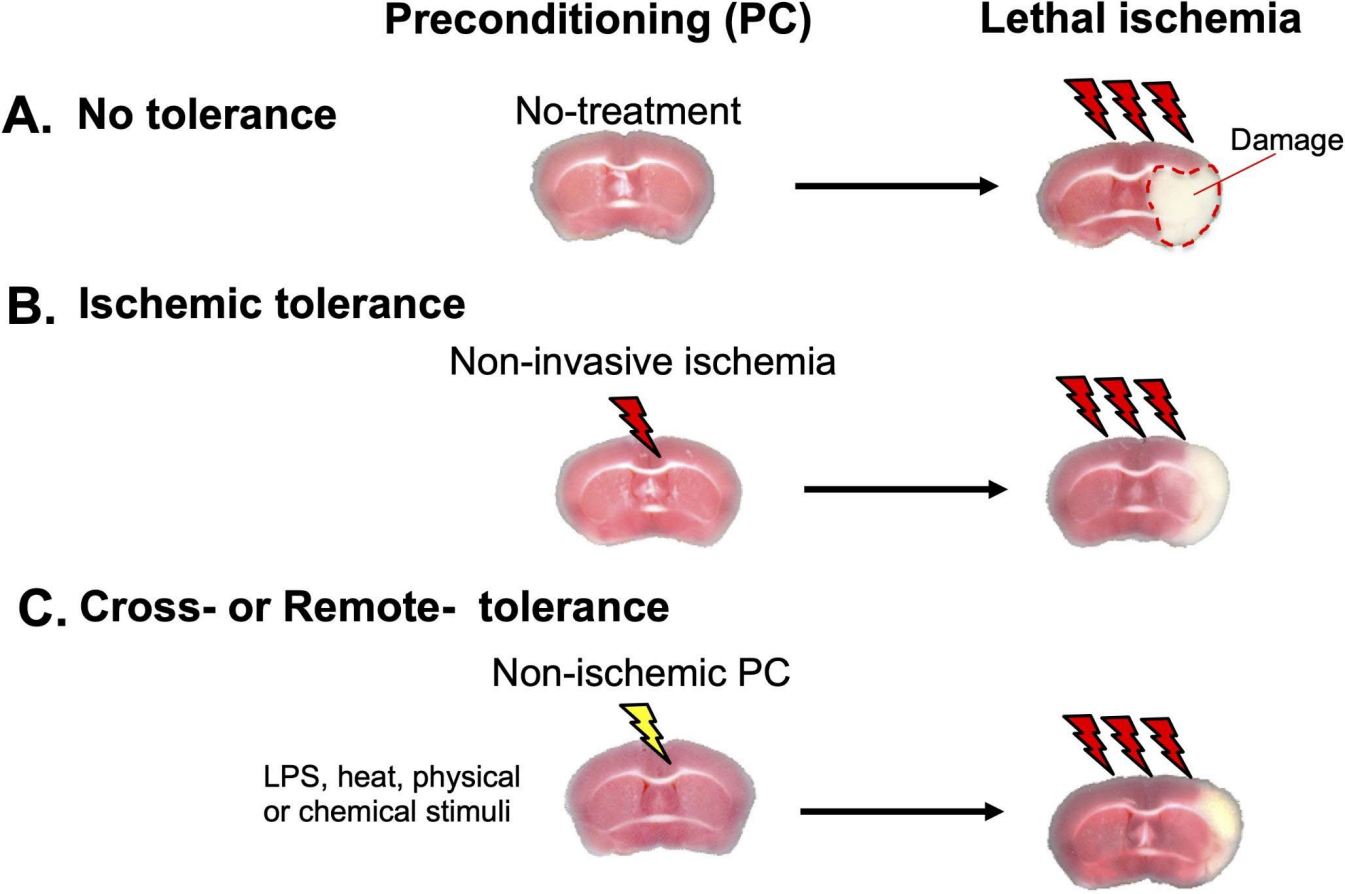
Ischemic tolerance (A) Brain tissues exposed to invasive ischemia (Lethal ischemia) develop severe neuronal damage and cell death (white area surrounded by dotted line). (B) If the brain experiences mild non-invasive ischemia as preconditioning (PC) prior to invasive ischemia, the damage induced by lethal ischemia is significantly reduced. This is termed ischemic tolerance. (C) PC that induces ischemic tolerance does not necessarily have to be ischemia; stimuli that mimic bacterial infection such as LPS, heat, physical stimuli, or chemicals can also induce ischemic tolerance. This is termed cross-tolerance or remote-tolerance

Although the development of stroke drugs has been difficult, research on “ischemic tolerance” has advanced significantly. Ischemic tolerance is a phenomenon whereby the brain, the organ most vulnerable to ischemia, acquires resistance to subsequent invasive ischemia after prior noninvasive ischemia (pre-conditioning [PC]), a phenomenon observed clinically and experimentally (Fig. 1). This has led to a great deal of intensive research, with an understanding of the molecular mechanisms of ischemic tolerance key to therapeutic strategies for stroke. Although several important molecules and intracellular signals have been reported to induce ischemic tolerance [4–7], most studies have focused on neurons and were based on the idea that tolerance is acquired by a neuron-autonomous mechanism. The brain is composed of neurons and higher numbers of glial cells, including microglia, oligodendrocytes, and astrocytes. Glial cells express neurotransmitter receptors, ion channels, and transporters, and release chemical transmitters called “gliotransmitters” in response to various stimuli [8]. Through this bi-directional communication between neurons and glial cells, glial cells regulate brain functions in a very immediate and active manner. Because glial cells are sensitive to environmental changes inside and outside the brain, when they sense these changes, they change rapidly and significantly. Thus, these glial changes are particularly important for the initiation and development of various brain diseases. Therefore, it is hypothesized that minor environmental changes in the brain, such as PC, are sensed first by glial cells, and that changes in their phenotypes may initiate a subsequent cascade leading to the induction of ischemic tolerance. It is well known that glial cells have protective effects on neurons, for example, suppressing astrocyte functions in an in vivo stroke model exacerbated neuronal damage [9]. We think that these findings support the present hypothesis. However, the aforementioned role of glial cells in ischemic tolerance has not been well studied.

In this article, we will introduce the latest findings on ischemic tolerance and glial cells, and explain glial ischemic tolerance, in which the brain is more resistant to stroke, with a focus on the roles of astrocytes [10, 11, 12,13].

## Brain Ischemic Tolerance

Ischemic tolerance was first discovered by Murry et al. in 1986 in a study of the heart [13]. Because the cardioprotective effect of ischemic tolerance is very strong and ischemic tolerance has been observed in many organs other than the heart, including the lung, kidney, liver, skeletal muscle, and the brain, which is the organ most vulnerable to ischemia, basic research on ischemic tolerance as well as practical research for clinical applications has been very active. The finding that ischemic tolerance was observed in vivo in the brain was first demonstrated in a Japanese study using a gerbil model of cerebral ischemia, and since then, many Japanese researchers have contributed to the development of this field [14] [15] [16]. The preceding stimulus to induce ischemic tolerance does not necessarily have to be an ischemic load. It is possible to acquire resistance to subsequent invasive ischemia even with the prior loading of a stimulus different from ischemia, such as hypothermia or lipopolysaccharide (LPS) mimicking infection, which is called cross-ischemic tolerance [16] (Fig. 1 C). Cross-ischemic tolerance can also be induced by chemical PC using chemicals such as 3-nitropropionic acid [17] or resveratrol [18]. For example, ischemic tolerance was induced in the brain even when a preceding load is applied to the hindlimb, which is distant from the brain, a phenomenon termed remote ischemic tolerance[19]. Regarding the therapeutic applications of ischemic tolerance, “prior light stroke loading” is not realistic. Therefore, the discovery of cross ischemic tolerance and remote ischemic tolerance is very important for research with a view to clinical applications. In addition, it is very useful for elucidating the molecular mechanism of ischemic tolerance, which is still largely unknown.

The initial studies of ischemic tolerance induction focused on neurons as neuron-autonomous mechanisms, with neuronal membrane stabilization, inhibition of neuronal excitability, and apoptosis inhibition being the main mechanisms proposed [16]. In addition, neurotrophic factors including heat shock protein (HSP) [6], brain-derived neurotrophic factor (BDNF) [7], erythropoietin (EPO) [4], vascular endothelial growth factor (VEGF) [5], and hypoxia inducible factor-1 (HIF-1) were identified as molecules involved in ischemic tolerance. Interestingly, these molecules are derived from neurons as well as glial cells. This suggests the importance of neuron non-autonomous mechanisms in the acquisition of ischemic tolerance, and their role is described below, with a focus on glial cells.

## Glial Cells and Ischemic Tolerance

### (1) Microglia

Microglia are immunocompetent cells in the brain that are activated in the early stages of trauma, infection, and various neurodegenerative and psychiatric diseases. Thus, studies on microglia have focused on their role as inflammation-induced injurious cells linked to the molecular pathogenesis of brain diseases. However, microglia have diverse functions, including the production of anti-inflammatory cytokines and neurotrophic factors such as BDNF, promotion of synaptogenesis, reorganization of neural networks by removing unwanted synapses, and removal of debris and unwanted substances by phagocytosis. Microglia are also cytoprotective cells that maintain brain homeostasis. As mentioned above, microglia are particularly sensitive to environmental changes and respond quickly to such changes. Indeed, the activation of microglia (strong Iba1-positivity) is the first step in PC. Importantly, it was demonstrated that pretreatment with endotoxins such as LPS induced cross-ischemic tolerance with a very strong protective effect against subsequent brain ischemia [20–22]. These findings suggest that microglia can sense LPS or LPS-related inflammatory microenvironments, and then change their phenotypes to protect neurons against subsequent brain ischemia. LPS-induced cross-ischemic tolerance is dependent on proinflammatory cytokines such as tumor necrosis factor-α (TNF-α), and the activation of Toll-like receptors (TLRs), both of which are present at high levels in microglia [20] [23]. More recently, the depletion of microglia by an antagonist of colony stimulating factor-1 receptor, a receptor essential for the survival of microglia, abolished PC-evoked ischemic tolerance in the white matter of the brain [24]. Signaling cascades and molecular mechanisms by which microglia induce ischemic tolerance via various TLRs are also being elucidated, with type I interferon (IFN) signaling and related molecules being involved. Furthermore, TLR4 and TNF-α were reported to be central in the mechanism of ischemic tolerance [25]. Therefore, microglia are likely to be key to inducing ischemic tolerance. However, how type I IFN-related molecules such as TNF-α induce ischemic tolerance and whether microglia alone can promote ischemic tolerance is unclear.

**Fig. 2:**
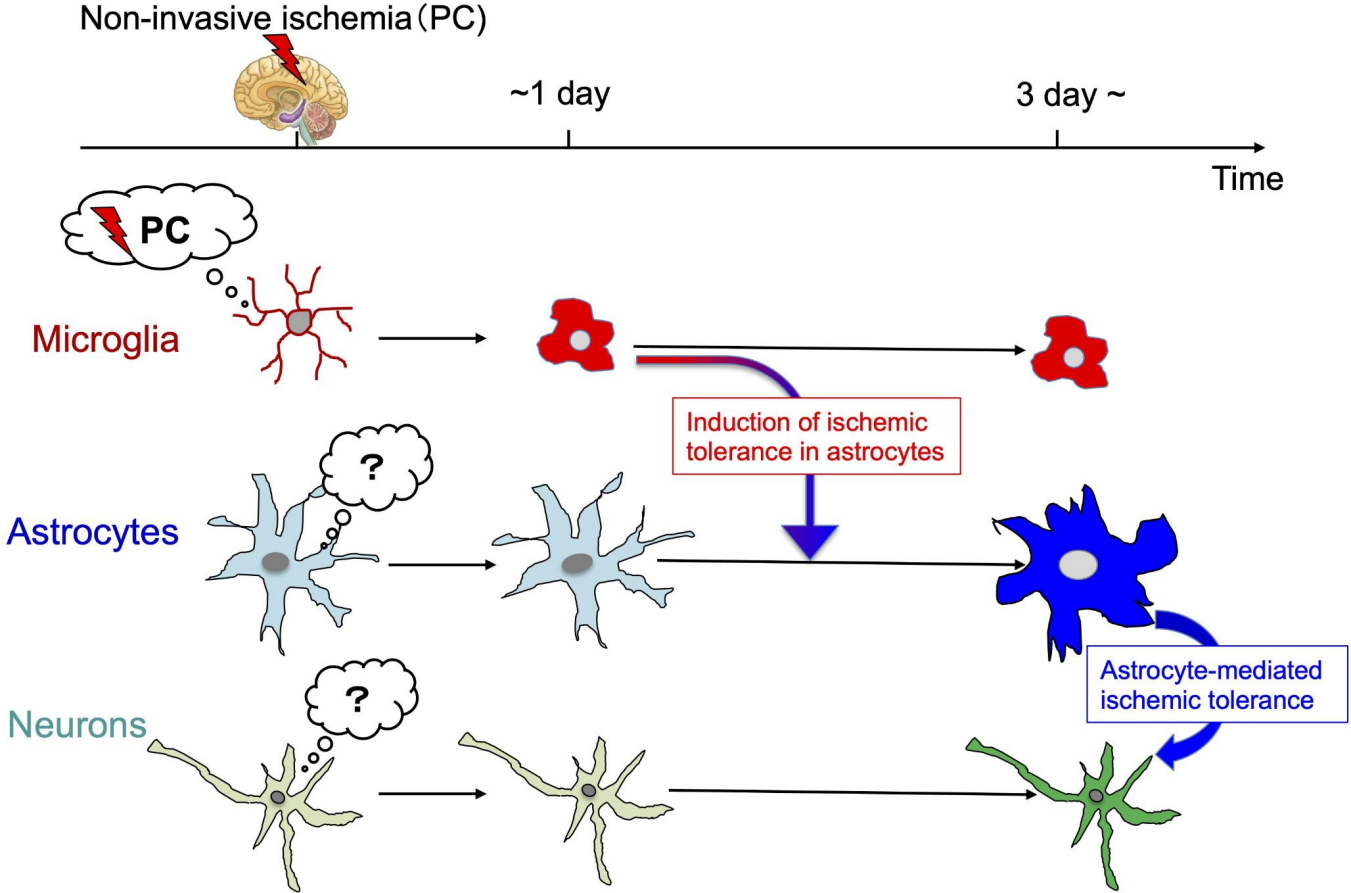
Sensing and responses to PC by glial cells and ischemic tolerance PC is a very mild load that does not damage neurons (non-invasive ischemia). Glial cells are sensitive to environmental changes inside and outside the brain, and microglia in particular are highly sensitive sensors that constantly monitor the brain. Microglia initially sense PC, which could be subsequently transmitted to astrocytes to form ischemic tolerance-type astrocytes. Microglia-astrocyte-neuron communication after PC is necessary for the acquisition of ischemic tolerance

In addition to directly affecting neuronal functions, microglia have attracted attention for their ability to regulate brain functions via microglia-astrocyte linkage. For example, ATP released from microglia secondarily regulated neuronal function by inducing astrocytes to release glutamate [26], and microglia that sensed traumatic brain injury secondarily promoted brain protective effects by transmitting this information to astrocytes [27]. Thus, microglia and astrocytes might communicate and share the roles, i.e., microglia may function as sensors to detect minute PCs and then astrocytes may act as execution factors to induce ischemic tolerance (Fig. 2). Next, we discuss the importance of astrocytes in the execution of ischemic tolerance.

### (2) Astrocytes

Astrocytes are the largest and most numerous type of glial cell. In addition to their classical roles of supporting neurons and processing waste products, they actively regulate the core brain functions including synaptic transmission, synaptic reorganization, blood flow, and energy metabolism. In a mouse model of middle cerebral artery occlusion (MCAO), noninvasive ischemia (brief MCAO) as PC activated astrocytes from 3 days after PC (which was slightly later than microglial activation) and they differentiated into highly glial fibrillary acidic protein (GFAP)-positive reactive astrocytes. The findings that (1) the spatiotemporal distribution of reactive astrocytes and brain regions where ischemic tolerance was induced after PC was well matched, (2) and that fluorocitrate (FC), an inhibitor of astrocyte activation, abolished ischemic tolerance [10], indicate that reactive astrocytes induced by PC are essential for the promotion of ischemic tolerance. In addition, PC-evoked ischemic tolerance was abolished in a GFAP and vimentin double knockout mouse, both of which are intermediate filaments and are known to be upregulated in astrocytes by PC [28], indicating that astrocyte activation was necessary for acquiring ischemic tolerance.

PC induced ischemic tolerance by enhancing glutamate transporter 1 expression, glutamate uptake, and suppressing excitotoxicity [29], indicating that the negative regulation of excitatory synaptic transmission is a mechanism of ischemic tolerance by astrocytes. Because ATP is a glial transmitter that has a central role in astrocyte-neuron coupling [30], we screened for ATP-associated molecules and found that the key molecule for ischemic tolerance. P2X7 receptors, which are associated with inflammation and cell death, are P2 receptors expressed in the normal brain, especially on microglia. However, a PC study revealed that P2X7 receptors were expressed more than 100-fold above normal levels in an astrocyte-specific manner, and their spatiotemporal pattern correlated well with the time course of astrocyte activation and acquisition of ischemic tolerance. Furthermore, the suppression of astrocyte activation with FC also suppressed P2X7 receptor upregulation, indicating that it is dependent on astrocyte activation. In addition, the acquisition of ischemic tolerance by PC in P2X7 receptor-deficient mice was completely abolished. Thus, PC transforms reactive astrocytes into “ischemia-resistant astrocytes” expressing the P2X7 receptor, suggesting that P2X7 receptor signaling is a prerequisite for the acquisition of ischemic tolerance[10].

**Fig. 3:**
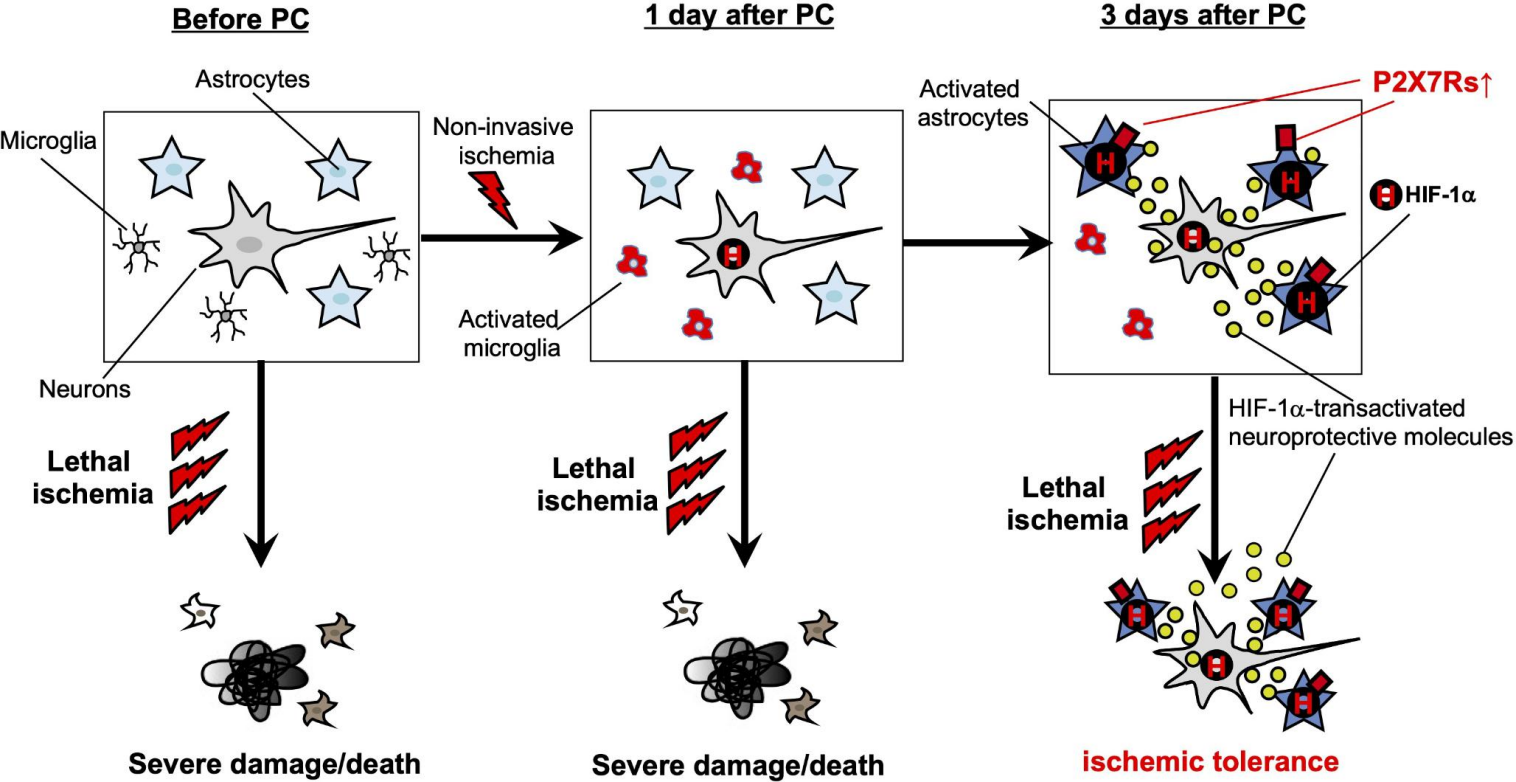
Mechanism of the induction of ischemic tolerance by astrocytes Left: Invasive load (lethal ischemia) before PC causes severe brain damage and neuronal death. Middle: One day after PC, microglia are initially activated, but at this time point, lethal ischemia still cause severe brain damage, and ischemic tolerance was not observed Right: About 3 days after PC, astrocytes become reactive and highly express P2X7 receptors, transforming into ischemia-resistant astrocytes. P2X7 receptor activation causes astrocytes to continuously upregulate HIF-1α expression. HIF-1α in astrocytes does not accumulate intracellularly in a hypoxia/ischemia-dependent manner, as in neurons, but is upregulated by a P2X7 receptor activation-dependent mechanism. Reactive astrocytes express the neuroprotective molecules EPO and VEGF in a HIF-1α-dependent manner, inducing resistance to subsequent invasive ischemia, i.e., ischemic tolerance

Next, we investigated which factors promoted the acquisition of ischemic tolerance and found HIF-1α was important. HIF-1α, a master molecule in the regulation of oxygen homeostasis, especially in neurons, where HIF-1α accumulates intracellularly during hypoxia, translocates into the nucleus and regulates the transcription of more than 100 important molecules. HIF-1α is homeostatically produced intracellularly, but is subject to rapid metabolism by oxygen-dependent degradative enzymes, and therefore it cannot function as a transcription factor at normal oxygen concentrations. However, in astrocytes, unlike neurons, HIF-1α does not accumulate by a hypoxia-dependent mechanism, but rather is upregulated by a P2X7 receptor-dependent mechanism [11]. In addition, P2X7 receptor expression in reactive astrocytes is persistent (several weeks), and thus HIF-1α expression is also persistent. This leads to a sustained increase in the HIF-1α-dependent transcription of various brain protective molecules including EPO [4] and VEGF [5], resulting in the production of high levels of neuroprotective molecules in the brain, and thus strong resistance to subsequent invasive ischemia (Fig. 3).

The importance of astrocyte P2X7 receptor and HIF-1α signaling in the acquisition of ischemic tolerance has been demonstrated. However, how astrocytes sense PC and the mechanism by which they enhance P2X7 receptor expression remains unclear: after PC, astrocyte activation and P2X7 receptor expression increase concurrently, and microglial activation precedes it. As mentioned above, microglia induce type I IFN-related molecules including TNF-α, which upregulates P2X7 receptor expression[31], during the acquisition of cross-ischemic tolerance induced by LPS[25]. This suggests that ischemic tolerance may be induced by communication between microglia and astrocytes, but the specific mechanisms involved require further study (Fig. 2).

### (3) Mechanisms of P2X7 receptor activation

The P2X7 receptor is a low sensitivity P2 receptor that requires very high ATP concentrations (0.3 mM or higher) for its activation [32]. However, PC does not induce high extracellular ATP concentrations ([ATP]o) [33]. It was reported that P2X7 receptors on peripheral immune cells can be sensitized by ADP-ribosylation, for which ecto-ADP-ribosyltransferase 2 (ARTC2) and nicotinamide adenine dinucleotide (NAD^+^), a substrate for ARTC2, have essential roles [34, 35]. Peripheral T cells express ARTC2 and P2X7 receptors, and P2X7 receptors are sensitized by ARTC2-catalyzed ADP-ribosylation. Thus, NAD^+^ can increase the sensitivity of cellular P2X7 receptors if ARTC2 is present. P2X 7 receptors on astrocytes might be sensitized by such a mechanism although astrocytes do not express ARTC2 under healthy conditions. However, ARTC2 is selectively upregulated by PC. The findings that NAD^+^ increased the sensitivity of P2X7 receptors to [ATP]o and the pharmacological inhibition of ARTC2 suppressed PC-evoked ischemic tolerance suggest that the ARTC2/NAD^+^-dependent ADP-ribosylation of P2X 7 receptors might occur in astrocytes. These sensitization mechanisms might explain how P2X7 receptors on astrocytes are activated by low [ATP]o and contribute to the formation of ischemic tolerance by PC.

## Future Directions

Recent findings on the importance of glial cells in the acquisition of ischemic tolerance were presented, with a focus on microglia and astrocytes. The importance of each glial cell is obvious, but we have also shown that ischemic tolerance may be acquired only when both cells communicate with each other. This review does not deny the results of many previous studies on the molecular mechanisms of ischemic tolerance that have been analyzed mainly in neurons. However, because inhibitors of astrocyte activation abolished ischemic tolerance, it is interesting to consider the possibility that the neuron-dependent ischemic tolerance acquisition mechanism is not neuron-autonomous, but rather neuron-non-autonomous as a result of communication with glial cells. In addition to microglia and astrocytes, and vascular systems [36] and oligodendrocytes [37] might also be involved in PC-induced ischemic tolerance. PC is non-invasive stimulation but some brain cells, especially microglia, sense PC and transmit its information to surrounding cells such as neurons, astrocytes oligodendrocytes and vascular cells. Therefore, future research on ischemic tolerance should not be limited to the framework of functional and molecular changes in individual cells, but should be viewed as an assembly centered on glia and realized as the sum of functional changes in the glial assembly. Spatiotemporal analysis using single-cell RNA sequences and functional analysis will be an important strategy for this research.

## Declarations

### Statements and Declarations

The data presented in this study are available on request from the corresponding author.

## Abbreviations

ARTC2: ecto-ADP-ribosyltransferase 2.
BDNF: brain derived neurotrophic factor.
[ATP]o: extracellular ATP.
FC: fluorocitrate.
GFAP: glial fibrillary acidic protein.
HIF-1α: hypoxia inducible factor-1α.
IFN: interferon.
MCAO: middle cerebral artery occlusion.
NAD^+^: nicotinamide adenine dinucleotide.
PC: preconditioning.
TLRs: Toll-like receptors.
TNF-α: tumor necrosis factor-α.
tPA: tissue plasminogen activator.
